# Influence of supercritical fluid extraction parameters in preparation of black chokeberry extracts on total phenolic content and cellular viability

**DOI:** 10.1002/fsn3.1645

**Published:** 2020-05-26

**Authors:** Jonathan Wenzel, Lihua Wang, Sebastian Horcasitas, Alyssa Warburton, Scott Constine, Anna Kjellson, Kirsten Cussans, Michelle Ammerman, Cheryl S. Samaniego

**Affiliations:** ^1^ Department of Chemistry, Biochemistry, Chemical Engineering and Applied Biology Kettering University Flint MI USA

**Keywords:** antioxidant, anti‐proliferative, *Aronia melanocarpa*, supercritical carbon dioxide

## Abstract

Black chokeberries (*Aronia melanocarpa*), deciduous shrubs of the Rosaceae family, are native to northeastern North America. Chokeberry fruits are cultivated to make jellies, juices, and wines. Black chokeberry pulp is rich in phenolics and other antioxidants and exhibits potential for health and food packaging benefits. Chokeberries’ in vitro antioxidant activity is among the highest values of all berries, though chokeberry extraction techniques frequently employ environmentally unfavorable solvents or are time‐inefficient. Batch extraction of antioxidants from chokeberry pomace using supercritical carbon dioxide with an ethanol modifier was used to examine the effects of plant loading, pressure, temperature, and percent ethanol by weight. Effects on total phenolic content (TPC) and the optimal conditions for extractions within these ranges are reported. Multivariate analyses reveal the following relationships of extraction conditions upon TPC: Temperature is directly proportional, percent ethanol by weight is inversely proportional, and chokeberry loads can be increased to enhance antioxidant activity, though not through a linear relationship. In studies involving 0.5 g plant load, the conditions 24.9MPa, 68°C, 90wt‐% CO_2,_ and 10wt‐% ethanol generated the highest TPC value, 3.42 ± 0.20 mg gallic acid equivalents/gram chokeberry. Chokeberry extracts displayed antiproliferative effects on the SKBr3 breast cancer line and the 52KO MEF line, although TPC was not predictive of cellular responses. HPLC‐MS data suggest cyanidin hexose and cyanidin pentose compounds as well as quercetin deoxyhexose–hexose as components of the more favorable extraction product that reflected a significant decrease in viability for the extract in comparison with ethanol control in the SKBr3 breast cancer line.

## Graphical abstract

Batch extraction of antioxidants from black chokeberry pomace using supercritical carbon dioxide with an ethanol modifier was used to examine the variables plant load, pressure, temperature, and weight‐% ethanol. Multivariate analyses were performed to determine relationships of extraction conditions upon total phenolic content. Chokeberry extracts displayed antiproliferative effects on the SKBr3 breast cancer cell line, and HPLC‐MS spectra at 254 nm suggest major components of anthocyanins cyanidin hexose and pentose compounds as well as the flavonoid quercetin deoxyhexose–hexose.

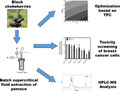

## 1 INTRODUCTION

Nutraceuticals, substances at the junction of “nutrients” and “pharmaceuticals” (DeFelice, 1995), have been used for years in treating disease. There has been a resurgence in popularity of dietary supplements, herbal remedies, and natural treatments due to factors such as a mainstream shift in preference toward natural over synthetic remedies, a desire to find more cost‐efficient treatments, and a need to find alternatives to medications with undesirable side effects (Nicoletti, 2012). Indeed, many proven medical treatments arose from natural plant‐derived products such as digoxin in treating atrial fibrillation, *Hypericum perforatum,*in treating depression, quinine in treating malaria, salicylates in treating fevers, and taxol in treating cancers (Aronson, 2017). Commercial production and distribution of nutraceuticals is a growing industry (Nicoletti, 2012), but there is growing inquiry for testing of natural compounds at all levels of drug development (Santini et al., 2018). Thus, there is an expanding market for cost‐efficient, environmentally friendly means of harvesting vast amounts of plant materials for nutraceutical benefit. Vital to this process is proper scientific assessment of true benefits and risks of supplements and plant‐derived treatments.

Plant polyphenols are a diverse group of compounds presenting a variety of human health benefits. Indeed, many health benefits of plants come from phenolic compounds including anthocyanins such as cyanidins, flavonoids such as quercetin, catechin, and resveratrol, proanathocyanidins, and phenolic acids. In the mixtures of which they are found in nature, there can be synergistic benefits of antioxidant, antimutagenic, antimicrobial, anticarcinogenic, and anti‐inflammatory properties, which are greater than those of the constituent parts (Juranić & Žižak, 2005; Katalinić et al., 2010; Rasouli, Farzaei, & Khodarahmi, 2017). The most prevalent subtype of polyphenols found in plants is flavonoids. Flavonoids, commonly attributed to the bitter taste and the yellow, orange, and red hues of fruits and vegetables, possess medical benefits such as anti‐inflammatory, antioxidant, anticancer, antiplatelet, antiviral, antiallergic, cardio‐protective, and cancer‐protective properties (Tanwar & Modgil, 2012).

Berries are particularly enriched in flavonoids and tend to have some of the highest overall amounts of phenolic compounds of all fruits. In berries, phenolic contents correlate highly with overall antioxidant activity. Anthocyanins, catechins, flavonols, and proanthocyanins are the predominant flavonoids in berries (Macheix, Fleuriet, & Billot, 1990). In a comparison of chokeberry, blueberry, cranberry, and lingonberry extracted with 80% acetone and 2% formic acid, chokeberries had the highest antioxidant values as assessed by three different estimators of antioxidant capacity: the oxygen radical absorbance capacity assay (ORAC), anthocyanin content through the pH differentiation method, and total phenolic content assays (Zheng & Wang, 2003). ORAC measurements reviewed by Kulling and Rawel suggested that chokeberries had the highest antioxidant capacities as measured by that assay in comparison with thirteen other berries, oranges, red and white grapes, and apples (Kulling & Rawel, 2008).

The *Aronia* berry, commonly called chokeberry, black apple berry, and rowanberry, is native to northeastern North America and the Great Lakes Region, and in the 1900s, this berry was introduced to Europe and Russia. The genus *Aronia* can be further categorized into the species *melanocarpa*, *arbutifolia*, and the hybrid *prunifollia*. Chokeberries are used as a component of fruit juice blends, jellies, teas, and wines, and as food coloring (Kulling & Rawel, 2008). Compared with other berries, chokeberries have a high content of polyphenols and sorbitol, and they display in vitro antioxidant activity, which is among the highest values of fruits (Denev, Kratchanov, Ciz, Lojek, & Kratchanova, 2012; Kulling & Rawel, 2008). Chokeberry's antioxidant activity has been attributed to a variety of *in vivo* mechanisms, including not only the traditional radical scavenging but also recharging antioxidant enzymes, inhibiting oxidant enzymes, preventing the formation of reactive oxygen and nitrogen compounds (ROS and NOS), and participating in signal transduction in response to oxidative stress (Denev et al., 2012).

Antioxidants hold great potential as nutraceuticals. Cellular tests suggest that antioxidants may help to combat destructive processes that result from accumulation of oxidative compounds because they block free‐radical damage and engage in signaling cascades as studied in various cell lines. Indeed, animal models and human clinical trials show multiple medical benefits of chokeberry administration in various forms (Chrubasik, Li, & Chrubasik, 2010; Denev et al., 2012; Kulling & Rawel, 2008). First, chokeberry juice may have value against autoimmune and inflammatory processes as it appears to decrease reactive oxygen species production and induced apoptosis in human neutrophils (Zielińska‐Przyjemska, Olejnik, Dobrowolska‐Zachwieja, & Grajek, 2007). Second, cellular studies involving complete or enriched chokeberry extracts show promising cellular death responses in several cancer cell lines including HT‐29 colon cancer cells (Olsson, Gustavsson, Andersson, Nilsson, & Duan, 2004; Zhao, Giusti, Malik, Moyer, & Magnuson, 2004), HeLa cervical cancer cells (Rugina et al., 2012), and MCF 7 breast cancer cells (Olsson et al., 2004). Third, chokeberry research suggests potential benefits to the cardiovascular system of rats and humans where diet supplementation with chokeberry products led to benefits in cholesterol profiles, blood pressure, and in cardiovascular endothelial cell restoration (Skoczynska et al., 2007). Fourth, anthocyanins isolated from chokeberries were able to decrease toxicity due to cadmium and carbon tetrachloride exposure and reduced amounts of heavy metals in the kidney and liver of rats (Kowalczyk et al., 2003; Valcheva‐Kuzmanova, Borisova, Galunska, Krasnaliev, & Belcheva, 2004).

A variety of extraction methods are employed today to harvest natural compounds from plants. A review of plant phenolic extraction methods at ambient pressure by Dai and Mumper demonstrates that various solvents including acetone, ethyl acetate, methanol, and ethanol are frequently utilized in varying combinations with water in traditional extraction procedures. (Dai & Mumper, 2010). In the ultrasonic‐assisted extractions (UAE) of dried chokeberries performed by d’Alessandro et al., it was reported that the phenolic yield of black chokeberry dramatically increased within the first hour of extraction with increasing temperature from 20 to 80°C (D'Alessandro, Dimitrov, Vauchel, & Nikov, 2014; d'Alessandro, Kriaa, Nikov, & Dimitrov, 2012). It is important to examine a wide breadth of temperatures, solvents, and extraction parameters when determining medicinal values of plant extracts.

Supercritical fluid extractions present an alternative method to extract medicinally relevant materials from plants. Supercritical fluids exhibit characteristics of liquids and gases. They exist above both the pressure and temperature conditions required for a substance to have a distinct phase boundary between the liquid and gas, and they are able to extract compounds faster than traditional methods (Sairam, Ghosh, Jena, Rao, & Banji, 2012). Superheated and supercritical fluid extractions have gained popularity due to their ability to extract without the use of organic solvents. Properties such as high diffusion coefficients of lipids and low viscosity actually increase rates of extraction while minimizing degradation. The most popular solvent used in supercritical fluid extractions is carbon dioxide, a nonpolar solvent, which offers protection against oxidation reactions (DeSimone, 2002). Recently, an extraction was employed on chokeberries using supercritical carbon dioxide with an ethanol modifier (Wozniak, Marszalek, Skapska, & Jedrzejczak, 2017), which used a partial factorial design where temperature, pressure, and ethanol concentration were varied, but solvent density was allowed to change with operating conditions. In contrast with the Wozniak studies, the parameters tested in the study described herein employed lower ethanol concentrations where solvent density was held constant, and pressure was allowed to vary with operating conditions. In addition for this paper, for some of the conditions selected, the solvent system was a binary supercritical fluid mixture of carbon dioxide with ethanol.

It is important to explore the best extraction conditions to harvest medicinal compounds from chokeberries. In this paper, we investigate a relatively nontoxic, batch extraction method to extract compounds from chokeberries by employing a solvent of supercritical carbon dioxide and an ethanol modifier (used to increase the dielectric constant (Schmidt & Moldover, 2003)) with different extraction parameters than previously employed by Wozniak et al. (2017). In this study, the variables temperature, percent ethanol, and mass of plant load are examined to determine the most ideal extraction conditions. The combination of temperature and pressure conditions that are above the carbon dioxide and ethanol mixture critical point may be identified using published experimental data and correlations (Pohler & Kiran, 1997). The total phenolic content (TPC) assay was employed as a preliminary screening method to compare concentration of probable phenolic antioxidant compounds obtained under varying conditions. Antiproliferative effects were determined for extracts of high, medium, and low TPC values on the SKBr3 breast cancer and *fkbp52‐*deficient mouse embryonic fibroblast (52KO MEF) control cell lines. HPLC‐MS analysis was performed to profile the most probable major components of the most antiproliferative extraction products toward SKBr3 breast cancer cells.

## 2 MATERIALS AND METHODS

### 2.1 Chemicals

The following reagent grade or greater chemicals were utilized: HyClone™ McCoy's 5A media (1.5mM L‐Glutamine, 2.2g/L sodium bicarbonate), Dulbecco's Modified Eagle's Medium‐high glucose (4.5g/L Glucose, L‐Glutamine, and Sodium Pyruvate) from VWR Life Sciences; Fetal Bovine Serum from Omega Scientific, Gibco™ Trypsin‐EDTA (0.25%), phenol red, Hyclone™ Phosphate‐Buffered Saline ((1X) 0.0067M PO4) without calcium, magnesium, phenol red, HyClone™ Trypan Blue Solution from Thermo Fisher Scientific, Optima LC/MS grade methanol and water, Optima LC/MS grade formic acid, molecular biology grade ethanol (200 proof) from Fisher Scientific; alamar Blue® from VWR, Gallic Acid monohydrate from Acros Organics; Folin–Ciocalteu reagent from Merck; Sodium Carbonate–Monohydrate from J. T. Baker Chemical; and ultrapure water produced on‐site. Nitrogen (99.998% purity) and carbon dioxide (99.5% purity) were from Praxair. Chemicals were used without further purification.

### 2.2 Plant material

Fresh, organically grown black chokeberries, *Aronia melanocarpa,* were obtained in western New York United States, during the 2017 growing season. Immediately following retrieval, the chokeberries were refrigerated. Subsequently, the chokeberries were destemmed, pressed, and the resulting pomace was frozen. The frozen pomace was then ground in a coffee grinder and stored in a nitrogen‐purged container at −20°C until time of use, up to one year later. The ground chokeberry pomace particle size ranged from 20 to 50 mesh.

### 2.3 Extraction method

Chokeberry pomace was extracted batch‐wise in a 24 ml, 2.54 cm outer diameter, 1.93 cm inner diameter, 316 stainless steel test cell capped with Swagelok® fittings using a previously described custom‐built batch extraction system (Wenzel et al., 2017). The extraction solvent was supercritical carbon dioxide with ethanol modifier. Temperature, ethanol weight fraction, and chokeberry loading were varied, with an extraction time of 60 min and a target solvent density of 0.76 g/ml. Since solvent density was held constant, the pressure must vary with temperature. Solvent loading was determined using the Peng–Robinson equation of state with Wong–Sandler mixing rules. Depending upon the experimental condition, for each experiment, between 0.25 and 1.5 grams of chokeberry pomace was weighed and placed into the test cell. Then, a predetermined amount of nitrogen‐purged ethanol was placed into the test cell. The test cell was then sealed, connected to the batch extraction system, and heated. Upon reaching the target temperature, carbon dioxide was fed into the test cell to the target pressure; then, the temperature was held at a constant for 60 min. Following this, the test cell was allowed to cool, the gaseous carbon dioxide depressurized, the vessel was opened, and the liquid extract suctioned out. The liquid extract was stored in a nitrogen‐purged, double‐sealed glass vial at 4°C in the dark. Prior to use in any assay, extracts were first centrifuged for 5 min at 1,690 *g* to remove solid residuals without concentrating the extract. Next, each extract was filtered with a vented Millex® 0.22um PVDF filter to remove any remaining suspended particulates for downstream applications of antioxidant and antiproliferative testing.

### 2.4 Total Phenolic Content/Folin–Ciocalteau Assay

The total phenolic content (TPC) assay or Folin–Ciocalteau assay is an electron transfer‐based colorimetric assay which quantifies reduction of a molybdotungstate indicator reagent in response to antioxidant activity of primarily phenolic compounds (Folin & Ciocalteu, 1927; Singleton & Rossi, 1965). Since the majority of antioxidant compounds in plants are phenolics, this assay is often used to estimate electron transfer‐based antioxidant activity in plants (Singleton & Rossi, 1965). The modernized version that was employed in this paper uses a 96‐well plate format (Ainsworth & Gillespie, 2007). The TPC assay was performed using the same methods as previously published by the team (Wenzel et al., 2017), though for the chokeberry studies, all extracts were diluted twentyfold prior to analysis and assays were performed in duplicate for three to four independent trials. Following the assays, statistical tests and analyses were performed as described in the Extraction Experimental Design section.

### 2.5 Extraction experimental design and statistical analysis

Two factors, temperature and ethanol content, were evaluated using a 2^2^ factorial design with randomization, with 2 replicates for corner points and 3 replicates for the center point. Temperature was varied from 50 to 68°C and ethanol content from 10 to 20 wt‐%. Chokeberry pomace loading for the factorial design was held constant at 0.5 g, and total solvent density was held constant at 0.76 g/ml. At least four independent analyses of duplicates for TPC were performed for each extraction sample. All experimental results were reported as mean values with corresponding standard deviations of assay measurements. The response for the factorial design, TPC, was evaluated by ANOVA analysis, where a p‐value less than 0.05 was considered statistically significant. Statistical analysis was performed using Minitab® version 16.2.0 statistical analysis software. Additionally, the effect of pomace loading was evaluated at 60°C, a solvent loading of 15 wt‐% ethanol, and total solvent density of 0.76 g/ml. Pomace loading was varied from 0.25 to 1.5 g, with each extraction performed in duplicate.

### 2.6 Cell Maintenance

52KO MEF cells, originally harvested by Tranguch et al. (2005) and SKBr3 breast cancer cells were a kind donation from Dr. Marc Cox at the University of Texas at El Paso Border Biomedical Research Center. Cells were maintained in an incubator at 37°C with 5% CO_2_ and split by addition of 1X 0.25% Trypsin‐EDTA (every 48 hr into 75cm^2^ tissue culture flasks (Cell treat®)) with either McCoy's 5a medium with for SKBr3 cells or Dulbecco's Modified Eagle's Medium for 52KO MEF cells, supplemented by 10% fetal bovine serum.

### 2.7 Alamar Blue® Assay

The alamar Blue® assay, first cited in 1993 for use in mammalian cells (Fields & Lancaster, 1993), is a highly sensitive, inexpensive, relatively nontoxic fluorescent quantification method often used in estimating cellular proliferation. It utilizes the weakly fluorescent property of the primary chemical resazurin and its conversion to highly fluorescent resorufin as an indicator of cellular metabolism by actively respiring cells. (Ahmed, Gogal, & Walsh, 1994; O'Brien, Wilson, Orton, & Pognan, 2000). SKBr3 or 52KO MEF cells were counted using Trypan blue per manufacturer's instructions and the BioRadTC20 Automated Cell Counter, followed by plating at 10,000 cells/well on Corning® Costar CLS3603 96‐well assay plates (black plate, clear bottom with lid, tissue cultured‐treated polystyrene). Following cell seeding, plates were incubated for approximately 16 hr at 37°C with 5% CO_2_. Then, media were replaced with new media containing 5% chokeberry extraction treatments, solvent, or media control. Cells incubated for 24 hr at 37°C with 5% CO_2_ followed by a wash in phosphate‐buffered saline. A 10% alamarBlue® solution was made directly with fresh media and was then added to the wells. Following a four‐hour incubation, plate fluorescence was detected (excitation at 540/35, emission at 590/20) using the BioTek Synergy HT microplate reader. Normalized values reflect a subtraction of fluorescence values of media alone, divided by the average fluorescence produced from live cell control. Assays were performed in replicates of 5 for at least 3 independent trials. Bar graphs were created and statistics analyzed using GraphPad Prism 8.1.2 (332) for Windows 64‐bit, Graphpad Software, La Jolla, California, USA, www.graphpad.com to reflect treatment responses expressed as normalized fluorescence in 52KO MEF and SKBr3 cells where error bars represent the standard error of samples. One‐way ANOVA analyses were performed with a Bonferroni's multiple comparisons test to compare ethanol‐treated cells versus live cells, ethanol‐treated cells versus treated cells for extract 1, 2, and 3, and cancer versus control cells of a given treatment. Statistical significance was assessed between different treatment groups’ results with *p* < .05. Finally, a direct comparison between adjusted fluorescence values of the SKBr3 breast cancer cell line and the 52KO MEF control cell line was performed by calculating the percent difference between their normalized fluorescence means for a given extract as follows:

$$\left|\frac{\text{mean normalized}\text{SkBr3fluorescence}-\text{mean normalized}\text{MEF52KOfluorescence}}{\text{mean normalized}\text{MEF52KOfluorescence}}\right|\times 100\%$$

### 2.8 HPLC‐ESI MS Analysis of Chokeberry Extract

Separation and mass/charge analysis of phenol were performed using high performance liquid chromatography (HPLC)–mass spectroscopy (MS) using an Agilent Technologies 1,200 Series HPLC instrument coupled with an Advion Expression Compact Mass Spectrometer. Before analysis, the sample was mixed with an equal volume of a solution containing 0.1% formic acid in 60% HPLC‐MS water and 40% HPLC‐MS methanol and filtered through a 0.45 µm Millex‐HV syringe filter.

A RESTEK Roc®C18 5µm 250 x 4.6 mm HPLC column was used for the separation. The flow rate was 0.5 ml/min. Peaks were detected using wavelengths of 254 nm, 280 nm, 350 nm, and 520 nm.

Separation was carried out using a formic acid/methanol gradient shown in Table 1. The mass spectrometer settings employed were the following: capillary temperature (250°C), capillary voltage (180 V), source voltage (20.0 V), source voltage span (0 V), source gas temperature (300°C), and ESI voltage (2,500 V).

**Table 1:** Experimental conditions of the separation gradient used in HPLC analysis

**Time (min)**	% of 0.1% formic acid in water	% of 0.1% formic acid in methanol
0	40	60
4	42	58
10	43	57
64	70	30
66	100	0
71	100	0

For general identification purposes, mass/charge (m/z) ratios and molecular weights of isolated peaks identified through ESI‐MS were compared against literature‐reported values of phenolic compounds previously extracted from chokeberries.

## 3 RESULTS AND DISCUSSION

### 3.1 Effects of supercritical extraction parameters on antioxidant potential of black chokeberry

Extracts of black chokeberry, *Aronia melanocarpa*, were prepared using supercritical carbon dioxide with an ethanol modifier as an extraction solvent. The effects of varying temperature and ethanol content were evaluated using a factorial design of experiments with replicates while holding total solvent density, chokeberry loading, and extraction time constant in the batch extractor. An independent set of experiments were also performed where solely chokeberry loading was varied. For the factorial design experiment, temperature was varied from 50 to 68°C and ethanol co‐solvent loading was varied from 10 to 20 wt‐%. Solvent density was held constant at 0.76 g/ml whereby the pressure was allowed to vary to maintain constant solvent density. The antioxidant potential for both sets of conditions was estimated with the total phenolic content (TPC) assay. Prior studies of berries show that the total phenolic concentrations correlate highly with overall antioxidant activity (Ga̧siorowski et al., 1997; Kähkönen et al., 1999; Prior et al., 1998; Wu, Gu, Prior, & McKay, 2004). The TPC assay was utilized in this study to estimate soluble phenolic compounds across the different extracts, though it is not to be taken as a comprehensive antioxidant study.

The effects of temperature and ethanol modifier percentage upon total phenolic content value for the factorial design experiment, represented by milligrams of gallic acid equivalent per gram of pomace (mg GAE/g), are displayed in Table 2. The maximum TPC value of 3.42 ± 0.20 mg GAE/g was extracted at 68°C and 10 wt‐% ethanol, which was the maximum temperature and minimal ethanol content evaluated. This also corresponds to the only condition evaluated where the temperature and pressure were both above the binary mixture critical point. While the solvent density was held constant, notably, this condition was also the highest pressure experiment at 24.9 MPa. The minimum TPC value was 1.32 ± 0.15 mg GAE/g, at the minimum temperature evaluated of 50°C and 20 wt‐% ethanol content, the maximum ethanol content. The pressure at this condition was the minimum at 12.2 MPa. For this study, pressure was only a dependent variable in the experimental design since solvent density was held constant. The analysis of variance of the factorial experiments evaluating the effect of ethanol content and temperature upon total phenolic content assay results of chokeberry pomace extracts is listed in Table 3. The main effects were statistically significant with *p* ≤ .05. The two‐way interactions between temperature and ethanol weight fraction were not statistically significant with *p* = .063, which may be due to experimental variability of temperature and carbon dioxide pressure, as well as TPC analysis of replicates. While the two‐way interaction is not statistically significant, given that *p* ≤ .1, a response surface analysis may yet provide some useful insight into optimal processing conditions, as well as providing a comparison to other studies.

**Table 2:** Antioxidant potential measured by the TPC assay for chokeberry pomace extracted using supercritical carbon dioxide with an ethanol modifier, 2^2^ Factorial design varying temperature and ethanol fraction. Solvent density was held constant at 0.76 g/ml

T (°C)	EtOH wt‐%	P (MPa)	TPC of Extraction Replicate 1 (mg GAE/g)	TPC of Extraction Replicate 2 (mg GAE/g)	TPC of Extraction Replicate 3 (mg GAE/g)
50	10	15.3	2.62 ± 0.17	2.46 ± 0.28	
68^a^	10	24.9	3.42 ± 0.20	3.34 ± 0.39	
50	20	12.2	1.32 ± 0.15	1.44 ± 0.18	
68	20	18.4	1.58 ± 0.77	1.92 ± 0.30	
60	15	19.8	1.92 ± 0.14	2.16 ± 0.17	1.90 ± 0.24

^a^ binary mixture of supercritical carbon dioxide and ethanol, ꝉsupercritical carbon dioxide with ethanol modifier.

**Table 3:** ANOVA of the effects of temperature and ethanol weight fraction upon antioxidant potential

Source	DF	*p*
Main Effects	2	.000
T (°C)	1	.001
EtOH (wt‐%)	1	.000
Two‐Way Interactions	1	.063
T (°C) x EtOH (wt‐%)	1	.063
curvature	1	.033

**Figure 1:**
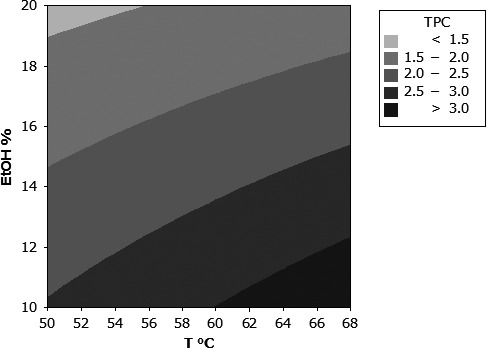
Contour plot for the effect of temperature and ethanol content upon total phenolic content for chokeberry pomace. Solvent density was held constant at 0.76 g/ml

**Figure 2:**
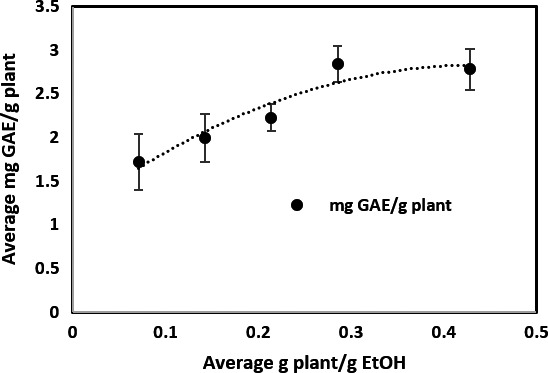
Relationship between total phenolic content of chokeberry extract and chokeberry loading in test cell where extraction conditions were average temperature 60°C, 15 wt‐% of ethanol, total density 0.76 g/mL, and hold time of 60 min

Figure 1 displays the response surface of the total phenolic content assay results for the interaction between ethanol fraction in supercritical carbon dioxide and temperature for the extraction of chokeberry pomace. As may be noted in the figure, as well as in Table 2, as the temperature increases, the total phenolic content assay value increases, which is expected. In addition, the total phenolic content also increases as the ethanol weight percentage decreases. Additionally, the effect of the amount of chokeberry pomace in relation to ethanol loaded in the extraction cell upon total phenolic content assay value was evaluated in Figure 2.

For the range of 50–68°C, it is notable that higher temperatures favored increased TPC assay yield. This is most likely due to influences of diffusivity; when temperature increases, so does the diffusivity of the extraction solvent. Additionally, as temperature increases, the matrix of materials that make up the chokeberry pomace relaxes, also enabling increased diffusion of compounds. This occurrence is limited by the decomposition temperature of the compounds extracted. A similar phenomenon has been observed in supercritical extraction of winery wastes, eucalyptus bark, walnut husks, and strawberries (Akay, Alpak, & Yesil‐Celiktas, 2011; Casas et al., 2010; Pinelo et al., 2007; Santos, Villaverde, Silva, Neto, & Silvestre, 2012).

In distinction from the conditions tested by Wozniak et al., density is held constant in the studies of this paper; therefore, pressures are equal to or higher than previously published, and the percent ethanol employed is only in the range of 10%–20% as compared with the range of 20%–80% from the other study (Wozniak et al., 2017). Due to maintaining a constant density in the studies, pressure did increase with increasing temperature, though it is not a direct variable in the study; the most optimal TPC value was obtained when the pressure was at its highest for the study, 24.9MPa. It is indeed notable that in supercritical fluid extraction of grape seeds and pomace, as well as with chokeberry, typically antioxidant potential will increase with increasing pressure, between 10 and 30 MPa, when total solvent density, carbon dioxide with ethanol, is not held constant. (Ghafoor, Al‐Juhaimi, & Choi, 2012; Murga, Ruiz, Beltran, & Cabezas, 2000; Pinelo et al., 2007; Wozniak et al., 2017).

In Figure 1 and Table 2, it is remarkable that as the ethanol content increases, the total phenolic content assay value decreases. Using supercritical carbon dioxide with an ethanol modifier, when pressure and temperature are held constant and ethanol content is increased, the solubility of natural phenols such as gallic acid, catechins, and quercetin increases (Chafer, Berna, Monton, & Munoz, 2002; Chafer, Fornari, Berna, & Stateva, 2004; Murga et al., 2000). Consequentially, studies of various plant species such as strawberries, guava seeds, eucalyptus bark, grape seeds, and grape pomace show that as ethanol content is increased, when temperature and pressure are constant, the total phenolic content of a variety of plant extracts increases to an extent (Akay et al., 2011; de Campos, Leimann, Pedrosa, & Ferreira, 2008; Casas et al., 2010; Castro‐Vargas, Rodriguez‐Varela, Ferreira, & Parada‐Alfonso, 2010; Santos et al., 2012; Yilmaz, Ozvural, & Vural, 2011). Again, it is important to consider the dependent variable of pressure in interpretation of the findings of this study. The difference between this work and the previously referenced studies is that for this work, total carbon dioxide/ethanol solvent density is held constant while ethanol content is varied; while for the referenced studies, pressure was held constant while ethanol content was varied. Due to the pressure–temperature–density relationship for supercritical fluid solvents, if density and temperature are held constant, pressure varies with changes in ethanol content. In contrast, if pressure and temperature is held constant, density varies with changes in ethanol content. Indeed, in the study evaluating the extraction of chokeberry pomace using supercritical carbon dioxide with an ethanol modifier by Wozniak in 2017, as pressure increased, the total phenolic content of the extract also increased (Wozniak et al., 2017). It is important to note that neither the range of ethanol content nor the density explored overlap with this work, yet, there is still overlap in the range of TPC values reported. For this study, since the total solvent system (carbon dioxide and ethanol) density was held constant, when ethanol content was increased, the pressure correspondingly decreased, as seen in Table 2. Based upon the experimental results, at the total constant density condition and ranges for this study, it is likely that pressure is a stronger indicator of extraction power for chokeberry pomace than ethanol content.

From Table 4 and Figure 2, the phenolic content assay value increased with increasing chokeberry load. The proportion of chokeberry to ethanol ranged from 0.071 to 0.429 on a mass basis. These experiments were performed using duplicate extractions, with the exception of the load of 0.5 g, which was in triplicate since it was the center point of the response surface analysis. The antioxidant potential was measured using the total phenolic content assay for three trials in duplicate. As chokeberry loading increased from 0.25 to 1.5 g, total phenolic content assay value increased from an average of 1.72 ± 0.32 mg GAE/g to 2.78 ± 0.23 mg GAE/g, though the relationship is not linear. While the relationship of these factors was not linear, it is important to note that for the conditions tested, it is possible to produce higher TPC values in chokeberry extracts by increasing the amount of plant matter added to the extraction cell for batch extraction.

**Table 4:** Antioxidant potential measured by the TPC assay for varying amounts of chokeberry pomace extracted using supercritical carbon dioxide with an ethanol modifier, T = 60°C, 15 wt‐% ethanol

Chokeberry (g)	Chokeberry/ Ethanol (g/g)	TPC Replicate 1 (mg GAE/g)	TPC Replicate 2 (mg GAE/g)	TPC Replicate 3 (mg GAE/g)
0.25	0.071	1.59 ± 0.23	1.86 ± 0.41	
0.5	0.143	1.92 ± 0.14	2.16 ± 0.17	1.90 ± 0.24
0.75	0.214	2.05 ± 0.23	2.41 ± 0.12	
1	0.286	2.86 ± 0.17	2.83 ± 0.24	
1.5	0.429	2.99 ± 0.18	2.58 ± 0.10	

### 3.2 Effects of black chokeberry extracts on cell proliferation

Berries and antioxidant components of berries have strong anticancer properties toward breast cancer cell lines (Aiyer, Warri, Woode, Hilakivi‐Clarke, & Clarke, 2012; Olsson et al., 2004). Some of the primary mechanisms of berry antioxidants in hormone‐dependent and hormone‐independent breast cancer cell lines may include targeting estrogen receptor signaling, targeting receptor tyrosine‐protein kinase erbβ‐2 [HER‐2] signaling, activating apoptosis, interacting with autophagy cascades, and modulating cell cycle regulation (Aiyer et al., 2012). The SKBr3 breast cancer cell line is classified as a human epidermal growth factor receptor 2 (HER2+) expressing cell line; it is deficient in expression of estrogen and progesterone receptors (Mota et al., 2017). Features of HER2 + cell lines are intermediate between luminal cell lines (representative of hormone‐responsive, less aggressive cancers) and basal cell lines (representative of hormone‐independent, more aggressive cancers) (Carey et al., 2006; Mota et al., 2017). The primary treatment used for patients expressing a similar molecular background to SKBr3 employs the monoclonal antibody Trastuzumab, but in cases of Trastuzumab resistance, there are few medicinal alternatives other than traditional chemotherapy (Carey et al., 2006; Maher, 2014). Ideally, compounds that show potent anticancer activity will have a higher IC_50_ (half maximal inhibitory concentration) in noncancer cells and will show minimal toxicity at the doses used to induce death in cancer cells.

**Figure 3:**
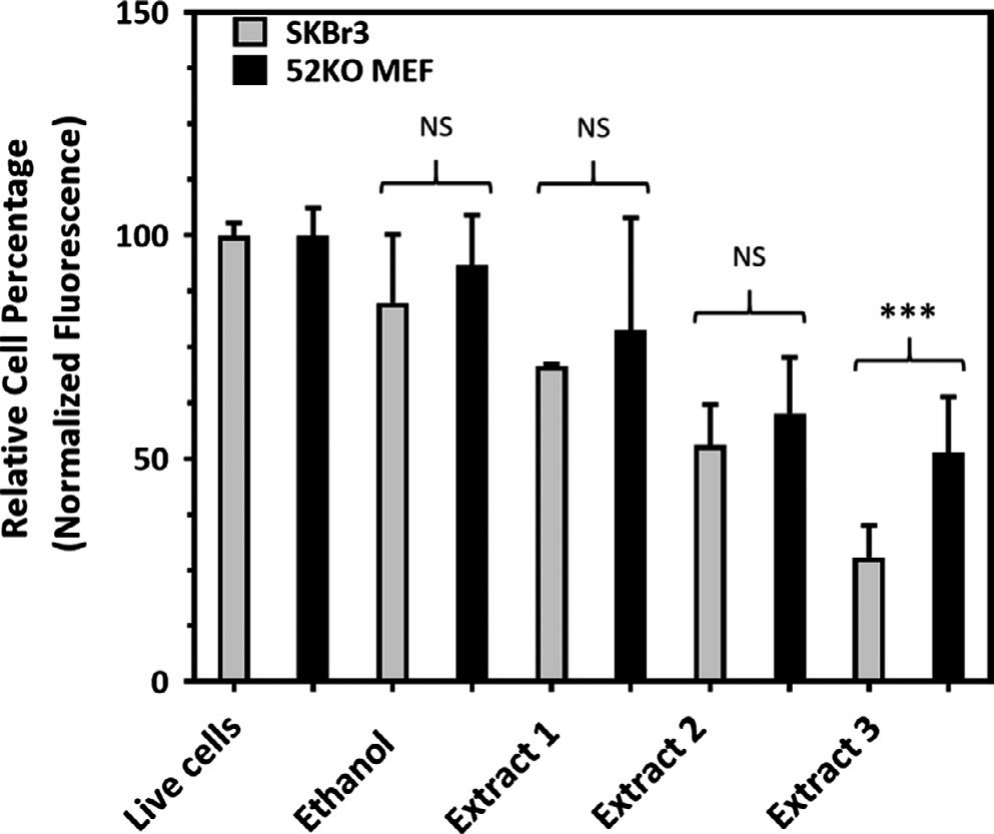
Chokeberry extracts exhibit antiproliferative effects on SKBr3 breast cancer and 52KO MEF cells. Error bars reflect standard error of the mean, and statistical significance reflects comparisons between cell lines for a given treatment. NS = not statistically significant (*p* > .025), ***indicates .0001 < *p*<.001

To this end, three chokeberry extracts were tested on both the SBKBr3 breast cancer cell line and the fibroblast cell line 52KO MEF. A fibroblast line was chosen as a control since fibroblasts are an active component of connective tissue found in the breast. Figure 3 shows the viability of SKBr3 and 52KO MEF cells using the alamarBlue® assay. Table 5 displays extraction conditions, TPC values, and alamarBlue® results for the three extracts on cell lines. For the extraction products tested, the total phenolic content assay values from chokeberry extracts ranged from 1.44 to 3.42mg Gallic acid equivalents/g chokeberry; extract 1 represents a mixture with a lower TPC value, extract 2 represents a mixture with a high TPC value, and extract 3 represents a mixture with a moderate TPC value. Extracts 1, 2, and 3 are listed according to increasing toxicity to SKBR3 and 52KO MEF cell lines—surprisingly, the mixture with the greatest toxicity had only an intermediate TPC value.

**Table 5:** Comparison between chokeberry extracts 1, 2, and 3’s extraction parameters, total phenolic content, and effects on cellular proliferation of SKBr3 and 52KO MEF cells

Extract	Solvent (%CO_2_/ %Ethanol)	Mean T (°C)	Mean P (MPa)	Loading mass of chokeberry (g)	Loading mass of solvent (g)	Antioxidant Value from TPC (mg GAE/g chokeberry) and σ^2^	SKBr3 % Proliferation from alamar Blue^®^ Assay (% Normalized Fluorescence) and *SEM*	52KO MEF % Proliferation from alamar Blue® Assay (% Normalized Fluorescence) and *SEM*
1	80%/20%	50	1743	0.5033	4.676	1.44 ± 0.18	70.77 ± 1.5	78.90 ± 3.2
2	90%/10%	68	3,500	0.501	2.353	3.42 ± 0.20	53.03 ± 1.2	60.06 ± 1.4
3	85%/15%	62	2,642	0.7521	3.511	2.41 ± 0.12	27.92 ± 1.1	51.36 ± 2.0

Note

σ^2^ represents standard deviation of the mean. *SEM* represents the standard error of the mean.

It was surprising to note that for the three extracts tested in Figure 3, total phenolic content of chokeberry extracts was not necessarily predictive of antiproliferative activity in SKBr3 and 52KO MEF cells. To test this theory further, extracts could be concentrated down relative to their TPC ratios prior to profiling in cellular studies, so that all extracts could be normalized to their TPC values and direct comparisons could be made. Concentrating extracts could also result in lowered ethanol content used in cellular assays, removing ethanol as a factor in inhibiting cell proliferation. From Figure 3, and Table 6, it is apparent that the ethanol solvent alone, given at 5% of the volume of the well, gave statistically significant decreases in proliferation in the SKBr3 cell line, though not in the 52KO MEF line. Even with the suboptimal ethanol: media ratio, the third extract offered promise in showing differentiation between the two cell lines and should be explored using an expanded range of concentrations to generate dose–response relationships in breast cancer and noncancer control cell lines. On the other hand, it is possible that an expanded dose–response curve in general for the two extracts might reveal a dose of the extracts which may have a higher distinction between the two lines. If, perhaps, the doses presented here represent concentrations that far exceed the IC_50_ values for the compounds in the cell lines tested, at a lower dose, there may be an even higher differentiation between the two lines.

**Table 6:** Statistical differences in cellular proliferation of SKBr3 and 52 KO MEF cells in response to named treatments

Comparison	SKBr3 Breast Cancer Line	52KO MEF Control Line
Ethanol: Live Cells	***	NS
Ethanol: Extract 1	NS	**
Ethanol: Extract 2	****	****
Ethanol: Extract 3	****	****

Note

NS represents not statistically significant (*p*>.05).

**** Indicates p <.0001

*** Indicates .0001 < *p*< .001

** Indicates .001 < *p*<.01

* Indicates .01 < *p* < .05

Cellular proliferation effects are most distinguishable in comparing the SKBr3 to the 52KO MEF line with Extract 3. This extract exhibited the greatest effects on cellular proliferation of the three extracts tested and showed a 45.6% decrease in proliferation compared with the fibroblast control. Extract 1 and 2 did not show statistical significance in toxicity between the cell lines. Because ethanol exhibited toxicity at 5% total volume, it was important to establish whether the effects seen by extracts were statistically greater than effects seen by ethanol treatments alone. Statistical comparisons of ethanol treatment versus live cell control and Extract 1, 2, and 3 individually are seen in Table 6. There is no apparent trend between any of the extraction variables (TPC value, extraction temperature, extraction pressure, and chokeberry load or ethanol percentage) and cellular proliferation.

In summary, it appears that Extract 3 exhibits a profile that warrants further investigation: toxicity to the breast cancer cell line SKBr3 with less toxicity to the fibroblast line. In the future, the dose–response profiles of total extract versus HPLC fractions of extract should be profiled. These could also be compared with synthetic standards of proposed compounds alone or in combination to determine the nature of the relationship between compounds and responses in the cell lines. It would also be interesting to explore the effects of the extracts and the mechanisms of cell death in other breast cancer cell lines such as MCF7 as a hormone‐responsive line and MDA‐MB‐231 as a hormone unresponsive, HER2 unresponsive, triple‐negative line.

### 3.3 Characterization of chokeberry Extract 3

**Figure 4:**
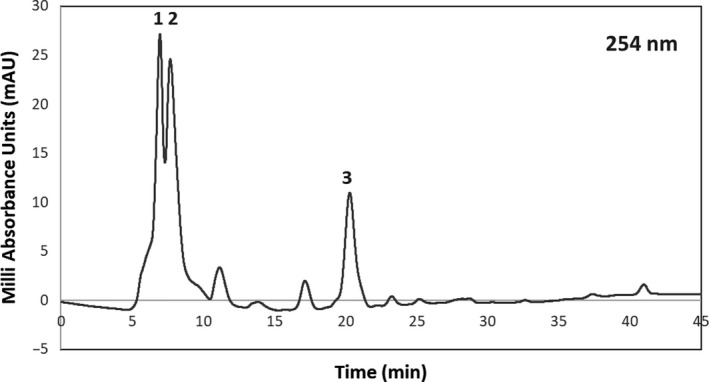
HPLC Profile of Chokeberry Extract 3 at 254 nm with three major peaks labeled: Peak 1 occurs at 6.967 min, Peak 2 occurs at 7.695 min, and Peak 3 occurs at 20.295 min

Due to the greater effect of chokeberry Extract 3 on proliferation of SKBr3 breast cancer cells over 52KO MEF cells, and the significant difference between the effects in SKBr3 over ethanol, Extract 3 was selected for HPLC‐ESI MS profiling. A study performed by Oszmiański and Wojdylo (2005) using sonication extraction of *Aronia melanocarpa* with methanol acidified with 0.1% HCl revealed a general distribution of the following types of phenolic compounds within chokeberry extracts*:* 66% polymeric proanthocyanins, 25% anthocyanins, 7.5% chlorogenic and neochlorogenic acids, and 1.3% flavonoids. The results of our HPLC analysis are displayed in Figure 4. Due to the solvent conditions described in this paper, proanthocyanins were not profiled in this study.

Analysis of chokeberry Extract 3 via HPLC‐ESI MS indicates the probability of multiple phenolic compounds. The mass spectra of the two overlapping peaks at 6 – 8 min indicated the presence of a cyanidin hexose and a cyanidin pentose, which is consistent with the finding of Zheng and Wang who had detected several cyanidin sugar conjugates including cyanidin galactoside (a cyanidin hexose) and cyanidin xyloside (a cyanidin pentose) by extracting chokeberry with acetone containing 0.2% formic acid (Zheng & Wang, 2003). In comparison with the supercritical extraction techniques used by Wozniak et al., which particularly probed anthocyanins, cyanidin‐3‐galactoside was the major compound, and cyanidin‐3‐glucoside was a minor component (Wozniak et al., 2017). This supports our finding of a cyanidin hexose. The fragmentation pattern of the mass spectra of the well‐resolved major peak at about 20 min indicates a high likelihood that it is a quercetin deoxyhexose–hexose conjugate, which is consistent with the finding by Häkkinen and Auriola (1998). In the negative MS spectrum, in comparison with the paper by Häkkinen and Auriola, the peak with a m/z ratio of 608.9 corresponds to the molecular ion [M‐H^+^]^‐^of a quercetin deoxyhexose–hexose conjugate, the 462.9 peak corresponds to the m/z ratio of a quercetin hexose ([M‐H^+^‐deoxyhexose]^‐^) fragment, and the 677 peak could be due to the sodium formate adduct of the molecular ion ([M‐H^+^+NaCHO_2_]^‐^). The positive spectrum of this component is consistent with the negative spectrum. There is a 610.9 peak in the positive spectrum, which corresponds to the m/z ratio of the molecular ion [M + H]^+^. The 303.0 peak corresponds with the quercetin fragment, the 465 peak corresponds with the quercetin hexose fragment, and the 486.9 peak could be due to the sodium ion adduct of the quercetin hexose. The fragmentation pattern is consistent with the finding of Häkkinen and Auriola. Three flavonol glycosides were found in the methanol/water extracts of chokeberry by Häkkinen and Auriola (1998): a quercetin deoxyhexose–hexose, a quercetin hexose, and a quercetin pentose. We detected the quercetin deoxyhexose–hexose as the main flavonol glycoside extracted from chokeberry using supercritical CO_2_ with ethanol modifier. A small amount of quercetin hexose was detected (HPLC peak at 11.1 min) but no quercetin pentose was detected. ESI‐MS analysis alone cannot tell the nature of the sugar units in the flavonol glycosides, and only the types of sugar units are reported here. Glucose and rhamnose were identified as the sugar units in the quercetin deoxyhexose–hexose conjugate detected by Häkkinen and Auriola using GC/MS of the HPLC fraction (Häkkinen & Auriola, 1998). Thus, our overall finding of anthocyanins cyanidin hexose and pentose as well as the flavonoid quercetin deoxyhexose–hexose are consistent with findings from others. Our HPLC findings seem to be consistent with literature but do reflect a more simplistic mixture of compounds (Oszmiański & Wojdylo, 2005; Wozniak et al., 2017). This contrast is likely due to the differences in extraction and HPLC preparation methods.

Considering the HPLC profile of Extract 3, it is of no surprise that there were anti‐proliferative results for the chokeberry extracts in the breast cancer cell line SKBr3 as seen in Figure 3. The suggested phenolic components of Extract 3, the most potent extract, were cyanidin hexose, cyanidin pentose, and quercetin deoxyhexose–hexose (Table 7); indeed, these types of compounds show promise as antiproliferative agents. In a review by Aiyer et al, where berry polyphenols were examined for their anticancer activities in various breast cancer cell lines, cyanidins were antiestrogenic and affected HER2‐dependent signaling by decreasing cell migration, autophosphorylation, phosphorylation of focal adhesion kinase (FAK) and p130cas adaptor protein, and association of the FAK/p130cas/HER2 complex. In the triple‐negative breast cancer cell line Hs578T, a caspase‐dependent apoptosis was elicited by cyanidins. Quercetin may exhibit either anti‐estrogenic or estrogenic activity in MCF7 cells, depending on the concentration administered. Quercetin can decrease tyrosine kinase activity, decrease expression of HER2, decrease phosphorylation of phosphoinositide 3‐kinases (PI3K) and Akt/Protein kinase B, and increase ubiquitination of HER2, in breast cancer cells (Aiyer et al., 2012). This information is particularly interesting because the SKBr3 line for which chokeberry exerts major effects relies on HER2 in part for growth; perhaps it is affecting HER2 directly or indirectly.

**Table 7:** Characterization of phenolic compounds from chokeberries using HPLC‐MS

Peak	t_R_ (min)	[M + H]^+^ (m/z)	Major Fragments/adducts positive spectrum (m/z)	[M‐H]‐ (m/z)	Major Fragments/adducts Negative Spectrum (m/z)	Molecular Weight	Probable Compound
1	6.97	448.9	287.0^a^, 163^b^			449 g/mol	Cyanidin hexose
2	7.69	418.9	286.9^a^, 162.9^b^			419 g/mol	Cyanidin pentose
3	20.30	610.9	303.0^c^, 464.9^d^ 486.9^e^	608.9	462.9^f^, 677^g^	610 g/mol	Quercetin deoxyhexose–hexose

^a^ cyanidin (M^+^)

^b^ fragment of cyanidin of ([C_9_H_7_O_3_]^+^)

^c^ quercetin ([M + H]^+^)

^d^ quercetin hexose ([M + H]^+^)

^e^ quercetin hexose‐Na^+^ adduct ([M + Na]^+^)

^f^ quercetin hexose ([M‐H]^+^)

^g^ quercetin deoxyhexose–hexose sodium formate adduct ([M‐H^+^+NaCHO_2_]^‐^)

## 4 CONCLUSIONS

Supercritical carbon dioxide extraction, using an ethanol modifier, is an effective means of extracting antioxidant compounds from chokeberry pomace. In this study, the extraction solvent density was held constant, while the ethanol content and temperature were varied, with pressure varying correspondingly according the relationships between pressure, temperature, and density for compressible fluids. The highest TPC value, 3.38 mg GAE/g chokeberry, was obtained at 68°C and 10% ethanol by weight, representing the highest temperature data point and the lowest percent ethanol by weight employed. Likewise, as chokeberry pomace loading in the extractor was increased, TPC values increased, reaching a maxima at 0.286 g chokeberry/g ethanol. In the alamar Blue®‐based cellular profiling of the chokeberry extracts, the extract prepared with 15% ethanol and 62°C caused a 72% decrease in SKBR3 breast cancer cellular proliferation and only a 49% decrease in proliferation in the control fibroblast line compared with live cell control. Comparative HPLC analysis suggested the antiproliferative agents cyanidin hexose, cyandin pentose, and quercetin deoxyhexose–hexose may contribute to the toxicity seen in the cell lines. In the future, mechanistic studies to assess means of cell death and profiling in a broader range of breast and control cell lines could be carried out to further characterize cellular responses.

## 5 ETHICAL REVIEW

This study does not involve any human or animal testing; there were no human participants from which to receive informed consent while conducting the study.

## CONFLICT OF INTEREST

The authors declare that they do not have any conflict of interest.

## Supporting information

Supinfo
